# Deaf Identity Under Pressure: Experiences of Deaf Persons in Iceland

**DOI:** 10.1093/deafed/enac049

**Published:** 2023-01-14

**Authors:** Kristinn Arnar Diego, Stefan C Hardonk

**Affiliations:** Centre for Disability Studies, School for Social Sciences, University of Iceland; Centre for Disability Studies, School for Social Sciences, University of Iceland

## Abstract

Over the past decades, changes in technology and policy have made developing and maintaining identity and community increasingly challenging for Deaf individuals. This is particularly obvious in Iceland, where the Deaf community is threatened in its existence. This paper reports on an empirical study that explores how Deaf individuals experience developing and maintaining a positive identity in various areas, including family life, working life, and social life. The study approaches deaf identities as multifaceted and dynamic, and particular emphasis is placed on the role of social interactions in identity-related processes. Qualitative interviews were conducted among members of the Icelandic Deaf community and analyzed using a phenomenological approach. The results indicate that social interactions within the Deaf community are a key factor in developing and maintaining Deaf identity. However, decreasing numbers in the Deaf community make its members explore other opportunities, and they experience being Deaf in Iceland as an insecure identity.

##  

Individuals’ identity is based on how they perceive and experience themselves, as well as the perspectives of others with whom they interact. Identities can be stronger or more fragile depending on how individuals manage to build on different aspects of social interaction. The formation of identity is in fact a process related to thoughts, emotions, and cultural factors, including attitude toward behavior (Sebastian et al., 2008). The identity of individuals is therefore both multifaceted and diverse, and this applies no less to deaf individuals. Moreover, deaf individuals have experience of different modes of communication and social connections that play an important role in shaping their identity. This paper reports on a study that examines how deaf individuals who define themselves as culturally deaf^1^ shape their identity through interactions with other d/Deaf individuals and hearing individuals. Particular emphasis is placed on the ways wherein individuals go about dealing with changes in the sparsely populated Deaf community in Iceland.

## Being Deaf in the Context of Increased Medical Intervention

Deafness has been defined in several ways. Moores (1978) has provided examples of biological emphasis, which leads to defining being deaf as when a person has such limited hearing that they cannot understand spoken language even if they use hearing aids. In this context, the perspective of medicine has been pointed out, which considers that those who have full bodily capacities are “normal,” whereas those who have bodies that deviate from biomedical norms are “defective” (Egilson & Pálmadóttir, 2006). Transposed to deafness, this suggests that the biological state of being deaf or hard of hearing is considered a challenge that creates barriers to participation in society. From this perspective, the emphasis is on stimulating hearing and speech (Hardonk et al., 2011a), and the systematic intervention of medicine in the lives of Deaf individuals has been described using the term “oralism” to emphasize the ideological effects of medicine that invalidate, ban, and eliminate sign language (Winzer, 1997) and consider spoken language as the best way to develop the minds of deaf persons (Lane, 1984).

However, other definitions have emphasized the interactions and community of those who use sign language in everyday life and have therefore been called a “cultural–linguistic perspective on deafness.” This perspective is not based on the biological characteristics of individuals but cultural heritage and social connections. That the so-called “Deaf culture” is valuable and that sign language as the language of Deaf people deserves to be respected and recognized, just like the cultural world of hearing people and their language. Deaf communities are real phenomena that have a long history of culture and language that is not based on the same assumptions as are prevalent in spoken language communities. Therefore, Deaf communities have appeared in a unique position toward the hearing community over time (Woll & Ladd, 2011). In view of Deaf people’s difficult experiences with the policies of oralism in the 19th and 20th centuries, the cultural–linguistic perspective assumes that Deaf individuals reject and oppose medical intervention. In this context, the Deaf community is defined as a minority group that demands recognition of sign language as a key element in the lives of individuals who define themselves as Deaf (Stefánsdóttir, 2005).

## Identities of Deaf Individuals

Identity is a key element in individuals’ experiences of participating in the community. Social identity theory emphasizes the role of values and attitudes related to a particular community group, including attitudes and behavior toward members within and outside the group (Schwartz et al., 2006). Furthermore, the possibility has been raised that social identity is associated with being a member of different social groups and that membership of these groups contributes to how individuals perceive themselves (Tajfel, 1981). According to this perspective, an individual’s social identity is related to positive feelings of belonging to a certain social group. From this perspective, Burke and Stets (2009) have described how deaf individuals seek out the community they believe they will be accepted, thereby creating opportunities for belonging (Burke & Stets, 2009). Dillon (1992) has pointed out the significance of communication in this process of identity development, that is, individuals need to be able to communicate socially with others in their own language to maintain identity and self-respect.

Building on social identity theory, scholars have commonly assumed that a deaf person’s identity is stable and related to either a medical or cultural perspective of deafness (Reagan, 2002). (Leigh 2009) has described these two distinct options in shaping the identity of deaf children. The first identity is centered on striving to be like a hearing person and use spoken language in communication. The individual subsequently seeks to participate in social interactions with the help of assistive devices, including hearing aids, which can be said to conform to a medical perspective on deafness aimed at “fixing hearing.” The second option according to (Leigh 2009) is to define oneself primarily from a cultural perspective as Deaf and form a Deaf identity, which associates positive meaning to sign language as their native language, and participation in the community of culturally deaf people who identify in a similar way. A well-known empirical study by Bat-Chava (2000) is an example of such a modernist approach that assumes that individuals’ identity belongs to a particular category out of a limited set of options. This study builds on a model of the development of culturally Deaf identities set forward by Glickman (1996) as a response to the medical approach to deafness. Glickman’s model relates Deaf identity not only to sign language but also to wider social and cultural factors, which leads to the definition of four possible identities. These identities are labeled “hearing identity,” “Deaf identity,” “bicultural identity,” and “marginal identity.” The study by Bat-Chava (2000) is an example of several studies that have operationalized this model using a set of questions aimed at measuring individuals’ attitudes toward the hearing community, Deaf community, sign language, and spoken language. Connected to this is the expectation that deaf persons develop the type of identity which offers experiences of self-esteem and life satisfaction (Bat-Chava, 2000; Hintermair, 2006, 2008; Leigh, 2009; Maxwell-McCaw & Zea, 2011). Other studies have used this classification to show that individuals with Deaf and bicultural identities are found to have more well-being, satisfaction with life, and self-esteem than those with hearing or marginal identity (Bat-Chava, 2000; Hintermair, 2008; Maxwell-McCaw, 2001). On this basis, scholars have recommended that deaf people connect with others who are deaf to gain a stronger identity (Leighet al., 2008). Moreover, they have pointed out the potential of connecting Deaf and hearing communities for strengthening Deaf people’s identity as bicultural (Bat-Chava, 2000; Glickman & Carey, 1993).

The classification of deaf individuals’ identities into a limited number of options has been criticized by scholars for lack of flexibility and contextualization. Rodda and Grove (2013) have highlighted the influence of different cultural and social factors in shaping identity. An example of an approach that considers historical and societal contexts to approach Deaf identity, in particular, can be found in Ladd (2005). He proposed the term “Deafhood” to describe how the identity of those who have defined themselves as Deaf from a cultural perspective is a process related to different aspects of the history and community of Deaf individuals. According to Ladd (2005), this process takes place within a Deaf community where individuals learn and shape the meaning of being “Deaf” through social interactions in sign language with other Deaf individuals. Although “Deafhood” considers different aspects and offers flexibility, Ladd has emphasized that it is in Deaf individuals’ nature to primarily identify with other Deaf individuals within their community. In addition, Deafhood is a process of developing self-respect and experiencing oneself on the same basis of existence on this earth as the hearing majority, which Ladd (2005) has assumed will lead to a stronger Deaf identity that has both a common core and different emphases between individuals. Another example of a response to criticism of research on the shaping of the identity of deaf individuals can be found in a study by McIlroy and Storbeck (2011). Their findings have suggested a process wherein individuals seek opportunities for belonging to a community. To the approach of a deaf individual’s identity that focuses primarily on culture and communication modes, McIlroy and Storbeck (2011) add the possibility for identities which flow between being hearing and Deaf. Other scholars have also highlighted the importance of considering the intersectionality of different aspects in the lives of deaf individuals (Chapple, 2019), not assuming that identity is stable (Skelton & Valentine, 2003). When examining the identity of deaf individuals from such a critical perspective, considering the context of individuals, it is important to examine the role of technological change.

## Context of Deafness Under the Influence of Technological Change

In recent decades, the identity development of Deaf individuals and the development of their community have taken place within a context of rapid technological change. This refers particularly to cochlear implants, which have become more effective and widespread, and, consequently, have affected the experience of deaf individuals and the status of the Deaf community (Cooper, 2019).

Although studies have shown that cochlear implants enable many, but not all, deaf individuals to learn spoken language (Sharma et al., 2020; Spencer et al., 2012), research has suggested diverse experiences of deaf individuals with cochlear implants when it comes to their identities. Cochlear implants have been controversial from the beginning within Deaf communities, and the intervention has been described by some as a continuation and reinforcement of oralism (Stefánsdóttir, 2005). According to Lane and Bahan (1998), cochlear implants reflect the “hearing norm”, that is, that everyone should hear and speak. Cochlear implants build on this norm, attempting to correct hearing capacity and allowing deaf individuals to hear. Stefánsdóttir (2005) has concluded that cochlear implants are incompatible with and are threats to the Deaf identity. Empirical research, however, has suggested a more complex picture. Studies based on a traditional classification of deaf people’s identity (e.g., Bat-Chava, 2000) have suggested that individuals with cochlear implants generally place more emphasis on being a hearing person in their identity. However, individuals aged 17–35 years with a cochlear implant in the study by Spencer et al. (2012) had a bicultural identity, that is, they shaped their identity in relation to being hearing and Deaf. Empirical studies by Marschark and Spencer (2009) and Szagun (2001) have concluded that individuals with cochlear implants had nothing to lose by learning both sign language and spoken language; therefore, everyone should be given the opportunity to make their own decisions regarding communication modes. Shook and Marian (2012), in another empirical study on the subject, have demonstrated that bilingualism improves intellectual skills and creates individuals’ opportunities for interacting and developing relations with both the Deaf and hearing communities.

The debate over the role of cochlear implants has been going on for several years, and scholars have questioned the simplifications in conceptualizing deaf identities that have often been used in empirical research and hinder the interpretation of results. Goldblat and Most (2018) have argued that cochlear implantation is only one factor in shaping the identity of deaf individuals, which takes place within a context that includes factors, including the biological status of hearing, education in mainstream or special schools, what modes of communication individuals have access to, and whether their parents are also Deaf. Furthermore, notwithstanding calls for recognizing the complexities in the development of deaf identities, traditional classifications have been highly influential in scholarship. In 1992, Lane highlighted the significance of Deaf individuals having access to Deaf education, clubs, arts, and sports where they meet other Deaf individuals and form relationships through sign language. A more recent example is the study on social connections of Deaf individuals by Gerich and Fellinger (2012), who concur with the study by Lane (1992), indicating that the number of connections with Deaf individuals affects the quality of life of Deaf people and that connections with other Deaf individuals enable Deaf people to face challenges. Traditional classifications of deaf identities have also permeated society. In Iceland, as well as in several parts of the world, public discourse has frequently been focused on the expected effects of cochlear implants on deaf children’s possibilities for participation. This has led to expectations regarding mainstreaming and integration of deaf children in hearing environments. However, Deaf communities have feared widespread adoption of cochlear implants and there has been little participation from people with cochlear implants in these communities. The widespread use of cochlear implants in children born deaf has created challenges on the individual and community levels. The lack of access to sign language may increase the risk of deaf children experiencing cognitive, social, and emotional issues, which have been referred to as “language deprivation syndrome” (Hall et al., 2017; Humphries et al., 2016). On the level of Deaf communities, the adoption of cochlear implants has led to fewer active members, potentially rendering those communities more vulnerable and putting further pressure on the sign language environment.

Noting that social interactions are key elements in the identity development of Deaf individuals, and that the context of deaf individuals in general, and those who are part of traditional Deaf communities in particular, has become increasingly challenging, this paper explores how Deaf individuals experience developing and maintaining a positive identity in various areas, such as family life, working life, and social life. More specifically, we report on an empirical study that aims to examine how the identity of Deaf people is shaped through social interaction and what paths Deaf individuals take to form and maintain a positive identity. We focus specifically on gaining an understanding of the identity of Deaf individuals from a critical perspective, that is, a perspective that assumes that identity is fluid and variable and is based on several aspects of people’s lives. The study was conducted in Iceland, which is interesting for several reasons. First, there is limited scholarly knowledge about this subject in Iceland, whereas the Icelandic Deaf community is under immense pressure. Consequently, insights based on the situation in Iceland can offer opportunities for transfer to other contexts. Iceland is a small country and this also applies to the Deaf community, which has been small throughout history. Moreover, hereditary deafness is uncommon; therefore, it is rare for several generations in a certain family to be Deaf. With the introduction of cochlear implants, the number of Deaf individuals has decreased even further in Iceland and the country can be seen as an example of the challenges that Deaf individuals face. The Language Committee on Icelandic Sign Language (Málnefnd um íslenskt táknmál, 2015) has drawn attention to the fact that the Deaf community is in danger of extinction in Iceland, notwithstanding the sign language is an officially recognized language. According to the law (Lög um stöðu íslenskra tungu og íslensks táknmál, 2011), the Icelandic State and municipalities must support Deaf culture, sign language, and education. Although this includes the provision of sign language education and interpretation, Deaf people face limited availability of free-of-charge sign language interpretation, and there is no legal right to sign language interpretation at work. In an op-ed for a national newspaper, the president of the Icelandic Association of the Deaf, Heiðdís Dögg Eiríksdóttir (Eiríksdóttir, 2013), has highlighted the significant consequences for Deaf people’s lives of the lack of access to sign language interpretation, which includes marginalization in family life and working life. The lessons learned from within this precarious context for being Deaf may be significant for other scholars, Deaf people themselves, policy makers, and professionals in other countries.

## Methods

The phenomenological research method of Vancouver School is best suited to increase understanding of human phenomena (Halldórsdóttir, 2003). This methodology seeks to return to the objects themselves, and the research should be critical and free from myopia. Metaphysical and scientific prejudices should also be avoided (Zahavi, 2019). The emphasis in this method is on the researcher gaining an understanding in collaboration with each participant and thus building a certain overall picture of what the experience being studied looks like from the point of view of this particular individual. Each participant in the study was considered a co-researcher, and the data collection took place through dialogs; the overall picture then compares the picture that was built regarding the next participant and so on until the overall picture of the phenomenon formed in the mind of the researcher (Halldórsdóttir, 2003).

The interviewees were three men and three women. Six Deaf individuals were selected using purposive sampling based on the condition that they all spoke sign language and considered sign language as their native language. This sampling criterion latter reflects the study’s focus on the experiences of persons who have culturally Deaf identities that relate to sign language and Deaf community. All six participants possessed language skills in everyday life in terms of Icelandic sign language and Icelandic, with Icelandic primarily being used by most participants as a written language. The age of the participants ranged from 25 to 30 years. This study was based on the experience of each individual interviewee and also on what could be called an atypical experience of it, and conscious work was performed to prevent the sample from being homogeneous (Halldórsdóttir, 2013).

Data collection occurred from September 2015 to September 2018 and was performed through interviews. Each participant was asked to sign an informed consent and anonymity form. At the end of the interviews, permission was requested from the interviewees to conduct another interview, if this proved necessary to gain a better understanding of the interviewees’ experiences. The decision to do so was made following the initial analysis of the data. If an aspect was discovered that required further information, the participants were contacted and asked for approval for another interview.

The interviews lasted on average for 1 h. The first author, who is proficient in sign language, spoke to all participants in Icelandic sign language. A framework was prepared for the interviews with these individuals, and everyone was asked the same questions. Initially, the researcher asked the participant a very broad question, and the dialog started. This is called an “open, non-standard interview” (Rees, 2009). The study followed these standard frameworks; however, it is necessary to emphasize that the qualitative methodology provided additions to the interview framework during data collection. In each interview, interview questions were used as guidance, such as “What does it mean to be Deaf?”, “As a Deaf person, what is your experience regarding social participation in the hearing community?” and “What factors strengthen and hinder the social participation of Deaf people in Iceland?” Subsequently, some sub-questions could follow previous questions, including “What do you think is involved in participating in the Deaf and hearing communities?” or “Describe incidents when you felt you were actively participating in the Deaf and hearing communities, and how did it shape you?”. Each interview was recorded with the consent of the participants. All interviews were subsequently transcribed verbatim in Icelandic.

This was followed by analysis of the interviews based on hermeneutics and constructivism. The program Atlas.ti (Scientific Software Development GmbH) was used for processing (coding) and data analysis, which started by the repeated reading of transcripts after which data were organized, categorized, and interpreted, leading to the identification of patterns of meaning. When interpreting data, we relied not only on the content of the interviews but also on how participants related their experience in context and what emphases they placed. Through a collaborative approach of data analysis and interpretation, the authors identified and wrote the main findings with respect to the aim of the study.

The Deaf community is a small community in Iceland, and it was decided not to describe the participants in detail to ensure confidentiality. The utmost confidentiality and anonymity were maintained when transcribing the interviews. To ensure anonymity, interviewees were provided names. The names provided to the participants were Sverrir, Óttar, Jón, Kristín, Snædís, and Vigdís.

Qualitative research methods were well suited to obtain information about participants. They were used to gain an understanding of behavior based on the experience of the person being studied. It was assumed that behavior was determined by how the interviewees interpreted their experience. In this qualitative research methodology, the research material was not approached from a rigorous theorical perspective, but examined starting from an explorative research question. The research question led the research, and the topics of our interview guide were developed alongside data collection. It was important that the individual belonged to the Deaf community and that they were ready to share their life experience. According to Vancouver School, data collection and analysis were simultaneously performed (Lofland, 2009). An interview framework with three main questions was used for all participants. According to the Vancouver School of Phenomenology, the individual engages in dialogs with the researcher and both are subsequently completely free to speak and listen (Halldórsdóttir, 2003).

In phenomenological research, although scholars are faced with the same study objective, they do not necessarily arrive at the same findings from their research. Tesch (2013) has highlighted that researchers have different experiences and paradigms and therefore see things differently. Therefore, the researcher must be constantly aware that the image of the phenomenon being studied may be different because the study of the experience of Deaf individuals can be varied in terms of environment, circumstances, and experiences. To increase the trustworthiness of this qualitative study, the authors were engaged in collaborative work aimed at acquiring data familiarity, understanding and categorizing meaning, and identifying patterns and findings. In addition, to present main findings from the study and inquire about their views and interpretations, the first author connected to Deaf persons in his social networks who had not participated in the study. The accounts of their own experiences further supported the reflection and interpretation of the results and consequently increased the trustworthiness of our study. This study was approved by the Icelandic National Research Ethics Committee (no. VSN-15-059).

## Results

The main results from the interviews are presented in this section. As the study was qualitative and the number of participants was six, drawing generalizations about the results was impossible; however, they provided certain indications. The following were the factors discussed in this section: Deaf identity in the hearing community, the role of the Deaf community in shaping identity, and future possibilities for Deaf identity.

### Deaf Identity in the Hearing Community

The interviewees wanted to use sign language because they can express themselves using their hands, whereas hearing individuals use spoken language to express themselves. These two different forms of communication prevented the interviewees from communicating with the hearing individual. Spoken language is typically used when interviewees need to communicate with a hearing individual; however, the interviewees in this study failed to perform due to deafness and can therefore be difficult for them. Sign language is their method of communication, which is as important as spoken language for the hearing individual. It is the basis of communication for Deaf individuals and their main method of communicating with others. If Deaf individuals feel that their sign language is not equal to spoken language, they may realize that spoken language is superior to sign language, which can have a negative effect on their Deaf identity.

As things stand today, interviewees are accustomed to yielding sign language to spoken language. However, due to deafness, they use Icelandic written language rather than spoken language for communication, and this can have several degrees of difficulty. Sign language and spoken language are different, and since Deaf individuals are unable to hear, they transcribe the written language as it is said in sign language. Several individuals are unsatisfactory with case inflection because they do not hear how the words are inflected. Consequently, several Deaf individuals do not want to communicate with the hearing individual because their written language often appears unusual, suggesting that this concern had a detrimental effect on their Deaf identity and hindered them in social interaction with the hearing individual. In this context, Kristín talked about obstacles she experiences in communication:

“I know I’m very bad at communicating. I’m not very good at articulating this and may not say things totally correctly or incorrectly. Sign language and Icelandic are not the same, which is why I may articulate things differently. Felt like they didn’t understand me.” (Kristín)

Several interviewees mentioned similar things about how difficult it is occasionally to communicate with individuals in spoken language.

All interviewees agreed that the Deaf community in Iceland is very small, which has consequently led to not selecting their friends.

The interviewees emphasized building a positive Deaf identity; therefore, it was important to be part of the community. Interviewees’ stories reflected different skills and experiences related to social interaction in different situations. However, they all believed that good and positive social interaction provides them with a positive Deaf identity. Sverrir, for example, speaks in this context of sign language as a key element in his life:

[It] is important for me to meet people so I can have a conversation. If I don’t see anyone, then I’ll be lonely, and I don’t like being lonely. There’s a reason God gave us sign language to talk to each other. (Sverrir)

In Sverrir’s experience, sign language is the best way to engage in social interactions with others and strengthen relationships. Sign language is part of the purpose of existence and prevents social isolation.

### Role of the Deaf Community in Shaping Identity

The interviewees described how they find places where they feel comfortable, which is in the Deaf community. They emphasized that in the Deaf community, they recognize the rules of communication and experience the security that comes with being able to speak sign language to other Deaf individuals. Kristín describes this in the following manner:

I mean, my only communication is with my deaf friends. These are the only people who understand me and I interact with... I can’t talk to someone who doesn’t know sign language. (Kristín)

Other interviewees described how it was reassuring to recognize that there are other Deaf individuals in this country who speak sign language. They experienced that when Deaf people with shared experience gather together, the group becomes cohesive, and everyone is ready to support each other.

I think it’s great. I mean I think it’s great and good, to know that there are other Deaf people here in Iceland. Here in Iceland we’re like one big family. Everyone who is Deaf meets up, and mostly at the Icelandic Association of the Deaf. The Association of the Deaf is our home, where we can be together. Social life is when Deaf people meet and share the same experience through sign language. (Kristín)

In Kristín’s words, the community appears very close-knit, and she compares it to family ties. However, although all interviewees experienced participation in the Deaf community as positive for their identity, it was obvious in the interviews that they perceived the Deaf community as far too small in Iceland and the group too dense. This is reflected in the fact that the interviewees expressed gratitude whenever more Deaf people join the Deaf community. Another manifestation is that the interviewees experienced a lack of choice in social connections and relationships. For example, Jón mentioned that in Iceland, it is common for Deaf individuals to not have a choice in with whom they wanted to develop friendships because the Deaf community is too small.

There’s only one other born in the same year as me, so he was the only one I could hang out with. I couldn’t talk to the other kids at school because they didn’t know sign language. So you could say that sign language was the only thing that connected us, which made us friends. (Jón)

Although Jón managed to build a friendship with his peer, his experience describes how he experienced that there were few other options for making a friend who knows sign language. Another interviewee, Sverrir, reports that although they do not have the same interests, he remains grateful for his group because this is a good group that meets regularly to have social interaction:

I like having Deaf friends... We always meet regularly and do something together. We don’t all have the same interests, but we don’t care because sign language is what matters. We like seeing each other and talking about something. (Sverrir)

Sverrir is content with being able to participate in an active group that uses sign language; however, his story also shows how he would have liked to develop relationships that consider his field of interest.

All the interviewees have been in the same group of friends for several years, and they talked about almost being able to write the biography of all their friends. This is such a small group of friends that everyone knows each other very well.

I like meeting Deaf people and being with them. But I’m always with the same group, always... I mean we’re totally friends and stuff. But if there had been many Deaf individuals, I doubt we would all have become friends. We’re very different, but because we were of a similar age, we had to be together. (Sverrir)

The limited opportunities for social connection in sign language indicate that Sverrir is an active participant in a group of friends, which he is not sure would be his group of friends under other circumstances.

This explains why the interviewees mentioned that they want more diversity in social interaction within the Deaf community in Iceland. When they rated communication within the community as monotonous, in some cases, they began to believe in going elsewhere, that is, looking outside the community and becoming more connected to the hearing community. Vigdís expresses participation in the hearing community along with participation in the Deaf community, which is small:... there are so few who are Deaf individuals here in Iceland... the community here is more like a hearing community and of course they want to be part of it. There may be a possibility that they are interested or curious about joining the community of the hearing, because they are bored in the community of the deaf because they are so few. The hearing community has much more to offer. (Vigdís)

It is interesting to note that Vigdís describes her point of view as the experience of everyone in the Deaf community, indicating that she experiences the search for social interaction outside the Deaf community not only as her own journey but as the reaction of the whole community to its small size.

Other reactions to the small size of the Deaf community that the interviewees discussed are related to emigration from Iceland to Deaf communities in other countries. All but one of the interviewees had lived abroad at one time. They all agree that Deaf communities abroad are considerably larger than they are used to in Iceland. Interviewees who went abroad described this change as though they had arrived at a new and larger home ground. Abroad, they got to know a more populous version of a Deaf community than they are used to in Iceland. Notably, participation in a larger community was described by the interviewees as an opportunity to experience a different sign language environment and a new dimension of social interaction.

A much bigger community. Yes, a much bigger community and more things to do. I felt more socially active. Honestly, it’s a big difference. There is just so much available abroad, but here it’s more always like.. . a small group, always the same people arguing and the same group who are friends. (Snædís)

It is worth noting how Snædís describes participation in Deaf communities abroad in a positive way and with an emphasis on possibilities. The description reflects the strengths and opportunities for her as a Deaf person. Conversely, she describes the Deaf community in Iceland in a negative way, as an uninteresting and weak basis for her Deaf identity.

Negative experiences related to the small size of the Deaf community were also discussed in relation to how difficult it can be to form an identity based on independence. For example, Óttar said that Deaf individuals in the community are constantly observing others:

It’s not fun finding out that everyone is watching me. This is my problem, and I don’t need everyone to tell me what to do. It’s annoying and stupid how curious everyone is about my issues. It’s none of their business. (Óttar)

In his telling, Óttar links his participation in the Deaf community to the lack of privacy and independence. He feels that being part of a small community often means that he needs to be ready to share all his issues with others and accept their views.

The interviewees who had lived abroad experienced a stronger Deaf identity. Overall, those who had participated in the Deaf community abroad enjoyed life there and found it comfortable to belong to a community that offered diverse social connections to Deaf individuals.

That’s why it is better to live abroad because there is much more freedom, and communication in sign language that strengthens you. You’re much happier as a Deaf person abroad than in Iceland. It’s easy to travel between Sweden, Norway and Denmark and meet other Deaf people. It’s much easier for these countries, but we’re just a small island far away and alone. (Kristín)

The Deaf identity that Kristín developed in the other Nordic countries seems to be stronger and offers more opportunities to connect with other Deaf individuals. However, she links being Deaf in Iceland to isolation and loneliness.

However, the interviews also discussed barriers to participation in a Deaf community abroad. It is frequently claimed that Deaf people find it easy to form strong bonds with other Deaf people across borders. However, it happens that not everyone has a positive experience of meeting Deaf people abroad. Óttar agreed with this and said that it could vary depending on cultures whether a Deaf person from Iceland achieves good social connections in a Deaf community abroad.

Deaf doesn’t mean that we’re all the same. I’m Deaf, but not everyone who is Deaf likes me. I’m a bit clumsy in human relations with the Deaf, but everyone here in Iceland knows me and knows how I am. But abroad, some Deaf people didn’t want to have anything to do with me, because I was always telling jokes. Then I realised that we’re not the same. I was so upset that the deaf were ignoring me that I broke down completely. (Sverrir)

It is interesting how Sverrir acquires a better understanding of how he is in the small Deaf community in Iceland, where individuals do not have the opportunity to ignore him and connect with others instead. However, in larger Deaf communities abroad, he believed that others had little interest in him, which he explained by emphasizing that he is not strong in social interactions.

While considering social connections, Óttar emphasized that factors, including interests and personalities, are more important when it comes to forming social connections, rather than people being Deaf or sharing cultures:

I like being around Deaf people, but not everyone. I mean... I don’t like everyone who is Deaf. Even though you’re Deaf, it doesn’t mean that I have to like you. I mean, if I were on a plane and I were Chinese, and then there was another Chinese person there, it doesn’t mean we have to be together. I’m human and I don’t like everyone. I want to talk to the Deaf, but then I also want to talk to those I like. Not everyone likes football. I want to talk to those who like the same things as me. (Óttar)

In other words, Óttar wants to form social connections with individuals he likes and has a connection with, and the fact that an individual is Deaf is not enough. This highlights the significance of having access to a community that is large enough.

One of the interviewees talked about the difficult experience of participating in a Deaf community abroad. Jón believed that he lacked strength in international sign language and the community shut him out owing to this. He experienced social rejection for the first time from Deaf people, and that was painful for him. It had the effect that he had doubts about his Deaf identity because the rejection came from Deaf people.

I once went to a Nordic festival for Deaf young people. I wasn’t good enough in international sign language or English, so the Deaf people were not interested in talking to me and kind of ignored me. I thought that this sucked so much that I only lasted for 2 days, but I was supposed to be there a week. Its bullshit that we’re all the same, and I’m not going to go to a Deaf festival again. (Jón)

How strongly Jón’s negative feelings are associated with his participation in a Deaf community abroad is worth noting. Instead of strengthening his Deaf identity, participation had the effect that he does not perceive any benefit in attending international Deaf festivals. For Jón, this experience destroyed the idea that “Deaf” means that all Deaf individuals are welcome in the community, and he became insecure about his Deaf identity, particularly in an international sense.

Despite these obstacles, the interviewees reported that to increase opportunities for social interaction, they opt to connect more with the Deaf community abroad. In addition, the interviewees described loneliness and lack of social connections with others, particularly individuals who use sign language, as obstacles.

I miss being able to do something on the weekends. When I was abroad, there was always something going on. There are so many deaf people that live there, and you can meet so many. But here, it’s completely different. There’s a lot of isolation. (Óttar)

Óttar described how the larger Deaf community abroad, wherein he participated, offers broad possibilities for social connections. According to him, being Deaf abroad is conducive to strengthening his social status and Deaf identity. He described this social activity as the opposite of his experience of participating in the Deaf community in Iceland, wherein he described himself as socially isolated.

Notably, the interviews revealed that rapid technological changes are related to the interviewees’ experiences of opportunities to form social connections within the Deaf community in Iceland and across borders. Moreover, they described their experience of how the Deaf community in Iceland has comprehensively adopted electronic communication, which in their opinion has led to a reduction in traditional social communication in person. They talked about how these changes have negative consequences for their well-being and how they perceive themselves as a Deaf person.

Before, we used to meet and talk. Much more like a club activity. We often had beer nights and things like that. But that has diminished tremendously... now most people are just at home online and looking at what this person and that person is doing. It’s just bad. That people avoid meeting and chatting, then it will become much more like loneliness. (Snædís)

Snædís was saddened that the opportunities to personally meet other Deaf individuals and as a group have decreased. She experienced emotions, including loneliness, when communication in the real world is sparse and electronic communication does not seem to completely replace personal communication.

### Future Possibilities for Deaf Identity

The interviews demonstrated the obvious apprehension of the participants regarding the future of social communication in sign language in Iceland. The effects of cochlear implants on the Deaf community were discussed; however, the interviewees perceived that fewer individuals participated in the community in parallel with the widespread use of cochlear implants. All interviewees stated that they were anxious about the future because the Deaf community in Iceland is declining owing to the low number of Deaf individuals. The interviewees described sign language as their security and mother tongue and considered the Deaf community as what keeps them alive. There, they enjoyed being around Deaf individuals who spoke sign language and understood other Deaf people.

It’s important to me to be with people who understand me. I don’t have to constantly try to explain what it is to be Deaf, and explain Deaf jokes and more... That’s why I feel most comfortable being around the deaf. That’s where the culture and community I live in is. Sign language is my mother tongue. (Sverrir)

Sverrir’s description of how the community and sign language are his life reflects how intertwined his identity is with participation and social connections within the Deaf community. The Deaf community provides him with meaning and security, and his account suggests that he experiences changes, such as an increased incidence of cochlear implants, as a threat to his own existence. Sverrir has shaped his identity as a Deaf person on the basis of the Deaf community, as he has known it since birth. The abovementioned quote shows how profound his fear of change is while the Deaf community is inevitably declining.

The fear of a dying Deaf community is great among the interviewees, and developments in the community seem to give them little hope. They said they feared that a growing number of deaf children will receive a cochlear implant, and the Deaf community would simply die out in Iceland. Interviewees reported that it was frightening to believe that the Deaf community in Iceland would be one of the first in the world to disappear. Such thinking evokes difficult feelings, including insecurity and helplessness. Interviewees perceive the increase in cochlear implants as a message from the hearing community that it is shameful to be Deaf, and that hurts their self-esteem. Vigdís concisely describes this as follows:

I fear that we are losing the deaf community. I mean... there are so few of us left and a child who is deaf is rarely born. Today, almost no deaf people are born. It happens, but then a cochlear implant is just put in and then it’s just bye bye. Sign language is dying out. The culture is dying out. (Vigdís)

Vigdís has no doubts about where she believes the community to which she is connected is headed. She is convinced that the Deaf community will disappear sooner. There is resignation in her words, and she does not seem to see any solution. Furthermore, anger is reflected as she experiences a lack of recognition from the hearing community. She interprets neonatal cochlear implantation in the sense that society finds it more desirable to develop a hearing identity than to be Deaf. Jón agrees by saying:

I would not want to see deaf children get a cochlear implant. I am proud to be deaf and I want to keep the Deaf community alive in Iceland. The National Hearing and Speech Institute is not helping individuals with cochlear implants to connect with the Deaf community in Iceland today. (Jón)

By his account, it is clear that Jón experiences that as soon as a decision is made about a cochlear implant, deaf individuals are directed to the hearing community by professionals. In other words, he believes that the cochlear implant and the services around it prevent the possibility of forming a connection between the children who receive the implant and those who are part of the Deaf community.

Moreover, fear of the future is reflected in the way that the interviewees talked about the next generation. They all said that if they had a deaf child, they would not get him/her a cochlear implant.

I can’t recommend a cochlear implant for myself, nor for my baby or children if they are born deaf. I think I’m used to being Deaf and using sign language and don’t see the point of a cochlear implant. I don’t know what it’s like to hear and will never know... why should I say yes to a cochlear implant for my children? (Kristín)

Here, Kristín talks about how she maintains her Deaf identity, as it has been shaped in connection with the Deaf community. From that perspective, as the technology has not been useful to her or the community, she perceives no reason to accept a cochlear implant for her child; on the contrary, it had the effect of weakening Deaf identity with the continuing decrease in the number of those participating in the community.

### Summary of the Findings

This study has examined how deaf individuals who define themselves as Deaf shape their identity through communication with other individuals, Deaf and hearing.

Interviews with Deaf participants indicate that in their identity, they place great emphasis on social interactions with other Deaf individuals and sign language. Interviewees experienced difficulties in forming a connection with the hearing community and talked about not being able to have deep, meaningful conversations. In most cases, such an experience led to a gap between the interviewees and hearing individuals, which can have a negative effect on a person’s Deaf identity. This encouraged participants to emphasize being Deaf and to group themselves even further together in the Deaf community. This is where the interviewees’ identity was formed first and foremost and where they were given the opportunity to strengthen their communication skills and social connections.

The Deaf community appears to be small, with close social ties that resemble family ties. Social communication in sign language was important for the interviewees and affected their identity. Interviewees relied on the Deaf community as their home ground, with culture, history, values and sign language enabling them to form strong social bonds and a positive Deaf identity. It was also noticeable that Deaf identity manifests itself differently among the interviewees and is never exactly the same.

The Deaf community is a key factor in the participants’ Deaf identity, but its small size and close connections within it were not only considered an advantage. Among other things, some found it difficult that a few Deaf individuals of a similar age are gathered into a specific group of friends. Interviewees experienced a lack of independence and diverse possibilities. Even though the interviewees did not feel that they had a lot in common with others due to for example different interests, they had to connect with them to avoid social isolation. As a result, it could be difficult to gain recognition of one’s privacy. Some of the interviewees responded by participating in Deaf communities abroad and experienced how it offered a more diverse social interaction in sign language and more Deaf events. Such connections with other Deaf communities abroad were an important part of the social network of some interviewees, which in some cases had existed for many years. In these cases, participation in well-populated Deaf communities abroad resulted in strengthening the Deaf identity of the individuals concerned. Obstacles to the formation of social ties in Deaf communities abroad related to culture, and sign language skills and interests were also discussed. Interviewees who had encountered such obstacles welcomed the understanding and security that the small Deaf community in Iceland has to offer. These results suggest that how Deaf individuals give meaning to a Deaf existence is related to circumstances and individual factors, for example communication skills and personality. Some interviewees found a strong Deaf identity in the Icelandic Deaf community, which is small and with social connections that resemble a family, whereas others needed participation in larger communities to form a positive Deaf identity.

Overall, the participants’ vision for the future was not promising regarding the possibility of social connections in the Deaf community in Iceland. Since the Deaf community is small and the number of people participating in it is decreasing, this finding is significant. Participants talked about the challenges faced by the community, and the dying out of, which would lead to further social isolation in the future. An interesting situation emerges: On the one hand, it turns out that the interviewees had a strong Deaf identity and sought out social connections with other Deaf individuals; on the other hand, being Deaf in Iceland may be an indication of an insecure identity, which is increasingly moving to the margins of society. Participants perceived that their expectations of a Deaf community wherein they had based their identity did not coincide with what they considered to be the prevailing views among parents of deaf children. Furthermore, our findings suggest that Deaf people in Iceland have no confidence in that they are envisioning a return to “traditional” Deaf culture and identity. Participants found it difficult to shape and maintain a Deaf identity in Iceland, and they saw few opportunities for future generations of deaf individuals.

## Discussion

This study aimed to gain insight into how Deaf individuals’ identities are shaped through social interaction and how they find opportunities to develop and maintain a positive identity. In this section, we draw conclusions based on the results of the study and discuss the value of the results for policy-making and research in the field of community participation of deaf individuals, both in general and with regard to Deaf people in particular.

Our findings indicate the diversity in Deaf identity development within a certain context. The small size of the Deaf community appears as an advantage to some; however, it is limited in potential for a strong identity for others. Moreover, the results suggest that social ties within the Deaf community can be experienced as too close. Going abroad provides opportunities that some need to maintain a positive Deaf identity, whereas others experience cultural barriers. We conclude that Deaf identity within the Icelandic context cannot be defined in one specific way; instead, individuals held different expectations and went different ways in shaping and maintaining a positive identity. Notwithstanding this diversity, there is common ground in the relationship between individuals’ struggles related to their Deaf identities and the circumstances within the Deaf community (see e.g., Foster & Kinuthia, 2003). These insights further develop what Ladd (2005) has described as individuals’ experiencing Deafhood in different ways, partly due to different social circumstances. This study illustrates the potential of placing issues of identity within a wider context and engaging with the complexities and contradictions in individuals’ experiences by considering factors beyond individuals’ preferred modes of communication and preferences regarding participation in Deaf and hearing communities (e.g., Bat-Chava, 2000). As such, being Deaf emerges as a continuous process wherein a person makes use of certain available resources. This leads to the conclusion that general and specific services used by Deaf people, such as schools and social services, need to consider the significance of offering diverse opportunities for being Deaf. Conversely, one-size-fits-all and essentialist approaches are not conducive to offering opportunities for diverse ways of shaping and maintaining a Deaf identity.

The pressure experienced by Deaf communities in Iceland and beyond is also reflected in the accounts of the participants in this study, who are faced with fewer opportunities for diverse social interactions with others who use sign language and identity in the same way as Deaf individuals. This study elucidated not only on the frequency of social interactions that take place in sign language but also the degree to which they correspond to the areas of interest of those who participate in them. Deaf individuals respond to a lack of social interactions in different ways, including seeking out Deaf communities abroad and participating in the hearing community. Interestingly, several participants pointed to cochlear implants as the main cause for the pressure on the Deaf community, and the adoption of the technology was interpreted as a rejection of their identity by the hearing community. Our study points to existential fears within the Deaf community in Iceland and feelings of being powerless to stop these changes. This has consequences for Deaf persons’ identities, which are increasingly linked to vulnerability, social isolation, and lack of future opportunities. This is related to the concerns raised by Ladd (2003) regarding Deaf people’s experience of the spread of cochlear implants among deaf children and adults. As a Deaf community moves into an existential crisis, the probabilities of deafness and sign language losing their positive meaning increase, and people feel disabled.

Notwithstanding these observations about the effects of cochlear implants on Deaf communities, the critical approach applied to identity in this empirical study also suggests the significance of diverse opportunities to strengthen the identities of Deaf individuals. In challenging situations, they are creative in their efforts to develop social relationships that provide them the opportunity to experience participation and recognition. Although cochlear implants as a technology will not go away, this study encourages considering ways to promote Deaf identities and expand the community of those who define themselves as Deaf in one way or another. Different actors in society share a responsibility to provide Deaf individuals with access to resources that enable them to develop and maintain an identity based on their interests, abilities, and circumstances. Although some Deaf individuals seek companionship and education outside the country, it is clear that the status of sign language requires to be strengthened at various levels in society. This includes access to sign language in the education system for all children—deaf and hearing. For Deaf people, sign language is not an aid, but a key part of their identity (Munoz-Baell & Ruiz, 2000). Therefore, increased sign language skills must be placed in the context of culture and participation, and it is significant to consider ways, such as individuals with cochlear implants who know sign language, to participate in the community of Deaf people. Generally, to date, increased sign language skills among those who do not belong to the Deaf community would be a way to increase social interactions based on common interests and strengthen the identity of Deaf people. Such a development requires a change in the policy of the authorities as well as a discussion within the Deaf community about who acquires access to a Deaf identity and the Deaf community. We will continue to discuss these two aspects.

Authorities for their part, need to review, on the one hand, what services are provided to deaf children and their families, and on the other hand, the context in which sign language is discussed. Studies have highlighted the influence of professionals (Hardonk et al., 2011b; Matthijs et al., 2012) on the decisions of parents of deaf children about communication modes and social participation, including in school. Authorities are responsible for early intervention and services for deaf children and their parents. In this regard, it should be noted that most parents of deaf children in Iceland are hearing. The Deaf community and the status of sign language can be strengthened by ensuring that cochlear implants, speech training, and rehabilitation do not reduce children’s access to sign language. Sign language is the basis of participation in the Deaf community, and it is therefore important that diverse deaf individuals can form social connections in a common language. In this way, possibilities are created for the social development of identity and a community where individuals with different interests, different abilities, and from different situations are connected. Such an approach of diverse possibilities for participation builds upon and expands what has often been referred to as “bicultural–bilingual identity” (e.g., Bat-Chava, 2000). Research indicates that bilingualism requires parents and professionals to use targeted methods in communication with deaf children; therefore, it is significant that support measures offer services in this area (Matthijs et al., 2018; Wilkinson & Morford, 2020). The institutional differentiation of early intervention services and services in the field of sign language, as is known in Iceland, is an obstacle to the implementation of this approach.

The challenges and opportunities discussed in this study have implications also for Deaf communities themselves, who need to find ways to become more diverse without sacrificing key elements of their identity. Developments in the struggle for the rights of minorities and the field of social science perspectives have led to the questioning of fixed definitions of societies and cultures (Meekosha & Shuttleworth, 2009). Moreover, this raises questions about how to define the Deaf community and Deaf identity and where the distinction lies between being Deaf and hearing (Woll & Ladd, 2011). It can be said that discussions about Deaf identity and the effects of cochlear implants on the lives of deaf people have been characterized by modernist approaches that do not allow for flow and flexibility. One aspect of this is related to the attitudes and expectations of Deaf individuals. Breivik (2001) has pointed out that communication between hearing and Deaf people can be complex and that hearing people can have difficulty connecting with the Deaf community. In this study, the results similarly indicate how Deaf persons regard people who received a cochlear implant at a young age as hearing individuals rather than Deaf, and there seem to be few possibilities for forming a social connection between them. In this context, Ladd (2005) has spoken about “audism” to highlight prejudices against those who do not meet certain criteria for Deaf identity, and Valentine (2007) has shown how deaf individuals can experience both support and rejection depending on the situation when a Deaf community has fixed ideas about what it means to be a Deaf person. This study adds to this discussion the importance for the future of Deaf communities of revisiting fixed beliefs of what constitutes being Deaf. If Deaf communities can find ways to include diverse individuals and open up Deaf identity for those using cochlear implants, Deaf communities may counter the decline in their membership. In addition, increased diversity may create opportunities for social interactions based on common interests.

In summary, this study underlines the need for efforts of societal actors that are grounded in a critical approach of how deaf identities are developed and maintained. This can support Deaf individuals to hold positive identities unaffected by fear of the future and the possible eradication of sign language in Iceland, but instead characterized by strength and active community participation.

This study deals with Iceland, which, as has been discussed, is in a specific position regarding the small size of the Deaf community. However, similar changes are occurring worldwide; therefore, this study contributes to a wider discussion. The voices of Deaf individuals must be heard in creating knowledge about the status and perspectives of all those who define themselves in some way in connection with being d/Deaf or hard of hearing.

## Endnotes

^1^In this article we use "deaf"to refer to the biomedical condition of having severely limited hearing capacity. We use "Deaf" to refer to deaf persons who identify as part of a cultural and linguistic minority in society.
